# Advancement of Peptide‐PAINT: Applications and Current Challenges in Super‐Resolution Imaging

**DOI:** 10.1002/smsc.70381

**Published:** 2026-08-17

**Authors:** Devi Prasanna Behera, Ritesh Sonar, Ahenjeeta Ghosh, Barun Kumar Maity

**Affiliations:** ^1^ Biophysical Sciences Division Saha Institute of Nuclear Physics Kolkata India; ^2^ Homi Bhabha National Institute Training School Complex Mumbai India; ^3^ National Institute of Science Education and Research Bhubaneswar India

**Keywords:** live‐cell imaging, peptide‐PAINT, single‐molecule localization, super‐resolution microscopy

## Abstract

Super‐resolution fluorescence microscopy has revolutionized the ability to probe biomolecular organization at the nanoscale in both fixed and living cells. Among various single‐molecule localization microscopy approaches, Point Accumulation for Imaging in Nanoscale Topography (PAINT) offers a conceptually distinct framework by leveraging transient probe–target interactions rather than fluorophore photo‐physics. Building on implementations such as DNA‐PAINT, Peptide‐PAINT has emerged as a versatile and accessible strategy for imaging protein organization with high specificity. This review provides a comprehensive overview of Peptide‐PAINT, covering its fundamental principles and recent methodological advances, including exchangeable, fluorogenic, and endogenous labeling strategies. We highlight its capability for multiplexed imaging and discuss emerging applications in live‐cell systems. Furthermore, we critically evaluate current limitations, including probe kinetics, orthogonality of peptide pairs, specificity, and intracellular delivery. Finally, we outline future directions for engineering robust, easy‐to‐implement Peptide‐PAINT platforms for quantitative and high‐throughput super‐resolution imaging.

## Graphical abstract

Peptide‐PAINT is rapidly emerging as a powerful single‐molecule super‐resolution imaging platform, enabling molecular visualization with ~5–10 nm localization precision. This review critically discusses its underlying principles, recent biological applications, technological limitations, and key challenges, while benchmarking its strengths and shortcomings against other single‐molecule imaging approaches and outlining opportunities for its future advancement.

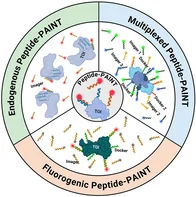

## 1 Introduction

The visualization of biomolecules in situ with nanometer‐scale precision has revolutionized cell biology and biomedical research. Within the cellular environment, biomolecules rarely act in isolation; instead, their functions critically depend on dynamic interactions as well as precise spatial organization, mostly at sub‐diffraction length‐scale. A conventional fluorescence microscope is diffraction‐limited, with a lateral resolution of ~250 nm and an axial resolution of ~500 nm [1]. As a result, key nanoscale features—such as protein clusters, macromolecular complexes (e.g., nuclear pore assemblies), and higher‐order protein assemblies—often remained unexplored for a long time. The advent of super‐resolution microscopy has enabled to overcome this barrier [2, 3, 4, 5]. Among various super‐resolution strategies, single‐molecule localization microscopy (SMLM) has emerged as a powerful and versatile approach. SMLM circumvents the diffraction limit by temporally separating fluorophore emission followed by localization of individual molecules with nanometer precision. Methods such as stochastic optical reconstruction microscopy (STORM) [6], photo‐activated localization microscopy (PALM) [7], and point accumulation and imaging in nanoscale topography (PAINT) routinely yield effective resolution on the order of 20–50 nm or better, enabling to decipher new insights into biomolecular architecture and interactions.

The central principle underlying SMLM is the stochastic blinking of fluorophores ensuring that only a sparse subset of fluorophores remains in bright state at any given time. This temporal separation prevents the overlapping of point spread functions (PSFs), resulting in precise localization of individual molecules [8]. In PALM/STORM, photo‐switching dyes or fluorescent proteins that undergo reversible or irreversible transitions between bright (“ON”) and dark (“OFF”) states achieve blinking through it. In contrast, PAINT relies on generating stochastic emission signals at the target sites through transient binding and unbinding of freely diffusing imager probes to the targets. The first demonstration of PAINT, shown by Hochstrasser et al., used a lipophilic dye, Nile red, that stochastically binds to large unilamellar vesicles (LUVs) and generates blinking of fluorescence signal [9]. Later, this approach was adapted by Jungmann et al. into DNA‐PAINT, where a short fluorescent oligonucleotide, known as imager strand, transiently hybridises with its complementary “docking” strand on the target [10, 11, 12, 13]. This innovation not only broadened applicability to protein imaging but also enabled remarkable localization precision, achieving sub‐5 nm. Furthermore, it has created the possibility of high multiplex imaging through programmable sequence design and sequential exchange of imager strands [14, 15, 16, 17, 18, 19, 20].

While DNA‐PAINT has been extensively developed and reviewed [21, 22], recent advances have introduced a peptide/protein‐based probe system, which is known as Peptide‐PAINT [23]. In this approach, DNA oligos have been replaced by peptide pairs, offering new opportunities for imaging protein targets with improved biological compatibility and flexibility [24, 25, 26, 27, 28]. Despite its promise, Peptide‐PAINT remains relatively unexplored compared to its DNA‐PAINT counterpart.

In this review, we provide a comprehensive overview of Peptide‐PAINT and its related methods. We discuss the fundamental principles including probe design and binding kinetics as well as its different variants developed to enhance its performance. These include fluorogenic Peptide‐PAINT (FPP), endogenous, and multiplexed Peptide‐PAINT. Furthermore, we highlight the underlying mechanisms, experimental implementations, and key performance characteristics of each approach, emphasizing their potential for quantitative, and high‐resolution bio‐imaging of complex biological systems.

## 2 Principles of Peptide‐PAINT

**FIGURE 1:**
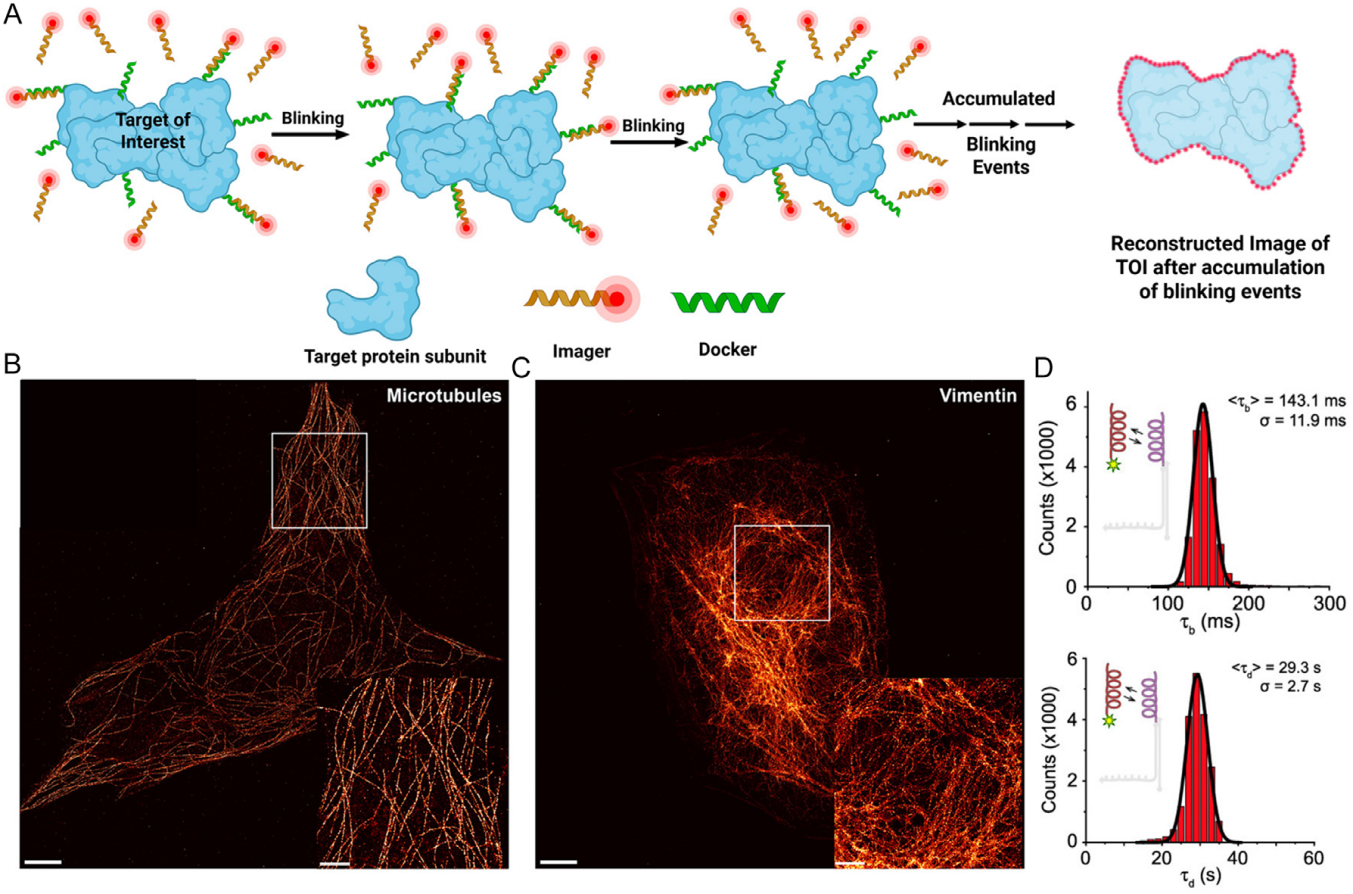
(A) Schematic describing binding–unbinding of fluorescent imager and docker during Peptide‐PAINT imaging; (B, C) super‐resolution image of cellular microtubules and vimentin filaments in fixed U2OS cells employing Peptide‐PAINT, scale bar: 5 μm; (D) histograms of binding (*τ*_b_) and dissociation (*τ*_d_) time between E19‐K22 with Gaussian fits (solid black line), highlighting the reversible interactions responsible for fluorescence blinking and high‐resolution image formation (Adapted from Eklund et al., Nano Lett., 2020).

Peptide‐PAINT is a single‐molecule imaging‐based super‐resolution microscopy method that relies on specific yet transient interaction pairs primarily of peptides. In this approach, one component of the pair, named as imager, is labeled to a fluorophore, while the binding partner, known as a docker, is associated with the target molecule. The docker is covalently appended to a target of interest using genetic engineering or noncovalently labeled through primary and secondary antibodies/nanobodies. In contrast, the imagers freely diffuse in the imaging solution and intermittently bind to the docker localized at the target sites. Each binding event results in a short burst of fluorescence signal before the imager dissociates from the target site, generating stochastic fluorescence “blinking” (Figure 1A). By carefully optimizing the imager concentration, probability of simultaneous binding events at the two spatially closed target sites is reduced so that only a few imagers remain bound at any given time. This ensures that fluorescence signals from individual molecules remain spatially separated, allowing their positions to be localized with high precision. The accumulation of these localizations over time enables the reconstruction of a super‐resolution image that reveals nanoscale organization beyond the diffraction limit of conventional optical microscopy.

The spatial resolution of a conventional optical microscope is fundamentally limited by diffraction and is approximately given by *λ*/2, as described by the Abbe diffraction limit [29]. Consequently, any two objects separated by a distance smaller than this limit cannot be distinguished and will appear as a single diffraction‐limited spot. However, the center of an isolated light source can be determined with much higher precision than the diffraction‐limit by fitting its intensity profiles [8]. This precision is fundamentally limited by the Cramer–Rao lower bound, which defines the best achievable accuracy for an unbiased estimator [30, 31]. However, experimentally, localization precision depends on several factors, including the number of detected photons, the width of PSF of the microscope, the effective pixel size of the camera, and background noise. A higher number of detected photons and lower background noise result in improved localization precision, enabling molecular positions to be determined with nanometer‐scale accuracy. Practical imaging conditions, additional noise sources degrade this ideal behavior, which has been described by Thompson–Larson–Web localization precision equation as shown [30].

(1) $$\text{Localization pecision}=\;\sqrt{\frac{s^{2}+\frac{a^{2}}{12}}{N}+\frac{8\pi s^{4}b^{2}}{a^{2}N^{2}}}\;$$

Here, “*s*” denotes the standard deviation of the PSF, “*a*” corresponds to the effective camera pixel size, “*N*” represents the total number of photons detected from an individual binding or blinking event, and “*b*” accounts for the background noise, detector noise, or out‐of‐focus signal. Collectively, these parameters determine the localization uncertainty and thereby influence the attainable spatial resolution in super‐resolution imaging experiments. At low photon counts, background noise plays a dominant role whereas at higher photon counts, photon counting noise becomes the limiting factor (Equation (1) ). We note that Equation (1) assumes approximately Poisson photon statistics. However, for an electron‐multiplying charge‐coupled device (EMCCD) camera, electron multiplication adds up an excess noise factor, which multiplies the value of localization precision by approximately $\sqrt{2}$.

In PAINT, with a given fluorophore, the number of detected photons can be effectively tuned by controlling the binding kinetics between the docker and the imager. Longer binding durations result in higher photon yields per localization events, thereby higher localization precision. However, this comes at the cost of increased imaging time, highlighting a fundamental tradeoff between localization precision and imaging time.

During a PAINT experiment, specimens are commonly imaged using highly inclined and laminated optical sheet (HILO) microscopy. In HILO illumination, an obliquely incident excitation beam generates a thin illuminated sheet (~6–10 µm) within a specimen, thereby restricting the excitation volume. This substantially suppresses the background fluorescence from freely diffusing imagers in out‐of‐focus regions and improves signal‐to‐background ratios, ultimately enhancing single‐molecule localization precision. Moreover, the reduced illumination volume minimizes photo‐bleaching compared to wide‐field epi‐illumination methods [32]. Consequently, a steady rate of imager binding and single‐molecule detections are maintained over prolonged acquisition periods, providing a notable advantage over photo‐switching‐based techniques such as STORM and PALM.

Beyond providing high‐resolution spatial information, PAINT has also been extended to quantitative molecular imaging through quantitative PAINT (qPAINT). It relies on stochastic binding kinetics between docker sites and diffusing imagers to estimate the number of accessible binding sites. Imager association follows bimolecular process characterized by association rate constant “*k*_on_” while dissociation is described by first‐order kinetics with rate constant “*k*_off_”. For a single docking site, the dark time (*τ*_d_) is primarily determined by the influx rate of imager (*ξ*) as described by Equation (2), where *C*_i_ is the imager concentration.

(2) $$\text{Dark time}\;\left(\;{\text{τ}}_{d}\right)=\frac{1}{\xi }=\frac{1}{k_{\mathrm{on}}*C_{i}}$$

For a region containing “*N*” equivalent and independently accessible sites, the effective imager association rate increases by *N*‐fold. Consequently, the mean dark time$\;\langle \tau_{d}\rangle$, obtained from intensity versus time trace of the region of interest decreases inversely with the number of available sites. Hence, the number of binding sites can be estimated as

(3) $$\text{Number of binding sites}\left(N\right)=\;\frac{1}{k_{\mathrm{on}}*C_{i}*\left\langle \tau_{d}\right\rangle }=\frac{1}{\xi *\left\langle \tau_{d}\right\rangle }$$

This kinetic framework enables qPAINT to infer molecular abundance independently of the number of localizations generated per binding event [12]. However, the quantitative accuracy of this approach relies on the assumption that individual docking sites exhibit comparable association kinetics and remain independently accessible to the imager. Steric constraints, heterogeneous local environments, or variations in docking‐site accessibility may alter the effective imager influx rate and consequently, bias molecular counting. These considerations are particularly relevant when extending qPAINT beyond DNA‐based docking strands to peptide‐ or protein‐mediated PAINT systems, where binding kinetics and target accessibility may be intrinsically more heterogeneous.

## 3 Variants of Peptide‐PAINT

### 3.1 Exchange Peptide‐PAINT

Exchange Peptide‐PAINT, first introduced by Eklund and coworkers, extends the PAINT concept by replacing DNA oligonucleotides with transient coiled–coil peptide pairs [24]. In analogy to DNA‐PAINT, the docker is conjugated to a secondary antibody targeting the protein of interest via a primary antibody, while the imager peptide diffuses freely in solution as mentioned before (Figure 1A). Unlike DNA oligos, peptide‐based systems lack intrinsic complementarity, making the design of suitable docker–imager pairs a major challenge. To address this, Eklund et al. employed a de novo‐engineered K3–E3 coiled–coil system, which has already been used for labeling a fluorophore to a membrane receptor in living cells [33], as a starting point. Such peptide pairs have been characterized by α‐helical heptad repeats that encode binding specificity through hydrophobic and electrostatic interactions. As the native binding time between K3–E3 was incompatible with PAINT requirements, binding kinetics were systematically tuned via C‐terminal truncations. Optimized variants—K22–E19, E22–K19, E22–K18—exhibited transient binding in the 60–200 ms range (Table 1), enabling efficient stochastic sampling during Peptide‐PAINT experiments. Notably, these peptide pairs demonstrated faster kinetics than DNA‐based counterparts, resulting in ~20% improved imaging efficiency. This strategy enabled super‐resolution imaging of filament proteins such as microtubules and Vimentin in chemically fixed U2OS cells [24]. However, the reliance on antibody‐based labeling renders the approach time‐intensive, expensive, and technically challenging, while introducing a substantial linkage error (~20–30 nm) that limits achievable spatial accuracy. These constraints underscore the need for next‐generation docker labeling strategies with improved specificity, reduced steric footprint, and simplified implementation (Figure 1B–D).

**TABLE 1:** Representative docker–imager pairs used in Peptide‐PAINT.

Sl. No.	Docker	Imager	Fluorophore	*K*_d_	On‐time, ms	Notes	Reference
1	K22	E20	Cy3B	—	242 ms	Exchange Peptide‐PAINT	Eklund et al., 2023 [26]
2	K22	E21	Cy3B	—	317 ms	Exchange Peptide‐PAINT	Eklund et al., 2023 [26]
3	P3(E29)	P4(K25)	Cy3B	—	259 ms	Orthogonal coiled–coil pair	Eklund et al., 2023 [26]
4	K22	E19	Cy3B	81 nM	—	Coiled–coil interaction	Eklund et al., 2020 [24]
5	K22	E18	Cy3B	1.7 µM	—	Coiled–coil interaction	Eklund et al., 2020 [24]
6	E22	CK19	LD655	—	220 ms	Live‐ and fixed‐cell imaging	Maity et al., 2023 [25]
7	101B	101A	mNeonGreen	—	50 ms	mLIVE‐PAINT	Bhaskar et al., 2025 [34]
8	KQTSV	PDZ3 protein	Organic fluorophore	670 nM	100–200 ms	Peptide–protein interaction	Gidden et al., 2023 [35]
9	SYNZIP18	SYNZIP17	mNeonGreen	1 nM	—	Live‐cell Peptide‐PAINT	Oi et al., 2020 [36]
10	MEEVF	TRAP4	mNeonGreen	300 nM	—	Live‐cell Peptide‐PAINT	Oi et al., 2020 [36]
11	PIPKIγ‐derived peptide	—	Cy3B	—	223 ± 98 ms	Endogenous Peptide‐PAINT	Fischer et al., 2021 [12]
12	PSD95 (PDZ domain)	KQTSV	Fluorogenic benzodiazole amino acid	—	100–300 ms	Endogenous Peptide‐PAINT	Moliner et al., 2023 [37]
13	DTWV	PDZ–mNG	mNeonGreen	0.1 nM	159 ms	Fixation‐compatible Peptide‐PAINT	Tas et al., 2021 [28]

Both Tas et al. and Maity et al. have mitigated the labeling issue by using a transfected docker where the transiently transfected plasmid encodes the DNA sequence of a target protein as well as a docker peptide at either its C‐ or N‐terminal. This enables expression of recombinant target protein appended to a docker peptide in the transfected cells only. We note that this approach cannot be executed if DNA oligo is used as a docker. Maity et al. have used the E22–K19 docker–imager pair (Table 1) and established the robustness of this approach by imaging various intracellular proteins in chemically fixed HeLa cells and outer membrane proteins in living neurons, and achieved ~10 nm localization precision. They have shown that Histone protein H2B is distributed in a clustered manner of size sub‐100 nm in the nucleus; longer filament of Vimentin is formed by longitudinal assembly of unit length filament (ULF) in mammalian cells. Furthermore, a high‐resolution image in live neurons reveals nanoscale distribution of AMPA receptors near synaptic cleft [25]. Although the approach has wider applicability, it suffers from the loss of binding efficiency of the docker due to nonspecific cross‐linking of lysine during chemical fixation by paraformaldehyde. It mandates an additional step of antigen retrieval. To address this, Tas et al. employed previously well‐characterized interacting pairs—the Erbin PDZ domain and a small peptide ligand, DTWV, which has low micromolar affinity and binding time ~159 ms (Table 1). Since DTWV is lysine free and not prone to nonspecific cross‐linking, it has been used as a docker and shows the binding time ~169 ms in chemically fixed cells, which verifies mitigation of fixative artifact. Thereby, this DTWV–Erbin PDZ domain pair has enabled to obtain high‐quality super‐resolved images of cellular vimentin, a filament protein, and TOMM20, a key import receptor localized on the outer membrane of mitochondria, under fixation condition in COS7 cells [28]. Quantitative assessment of the image quality reveals that single‐molecule localization precision is 15.4 ± 8.5 nm and resolution is 53 ± 4 nm. Additionally, negligible nonspecific interaction between PDZ‐mNG and fixed cellular sample makes this system favorable for Peptide‐PAINT. However, continuous fluorescence background arising from freely diffusing docker limits the maximum concentration for PAINT experiments. HILO illumination, which is being primarily used in PAINT experiments, reduces background by spatially restricting excitation rather than eliminating fluorescence from the unbound imagers. Consequently, achievable imager concentration remains limited by background fluorescence, creating a fundamental tradeoff between binding frequency and image contrast. This concentration‐dependent constraint lengthens the image‐acquisition time, which indirectly becomes the limiting factor for imaging speed.

### 3.2 FPP

**FIGURE 2:**
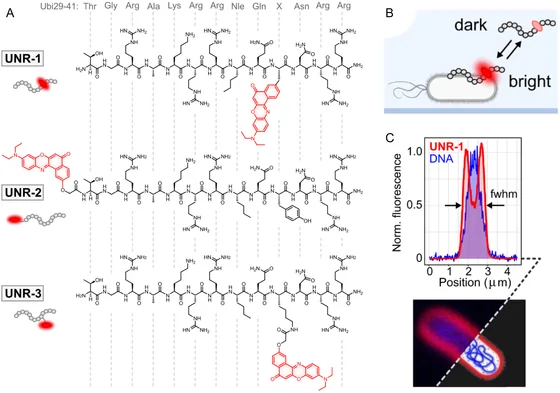
(A) Structures of nile Red–labeled ubiquicidin peptides illustrating different conjugation strategies to incorporate unnatural amino acid; (B) schematic of fluorogenic Peptide‐PAINT using ubiquicidin peptides; (C) normalized fluorescence intensity profiles derived from two‐color confocal fluorescence images of bacteria co‐labeled with UNR‐1 (red) and Hoechst 33342 (blue); FWHM denotes full width at half‐maximum (Adapted from Weiss et al., Bioconjugate Chem., 2024). Reprinted with permission from Ref. [39]. Copyright 2026, American Chemical Society.

FPP is an emerging variant which eliminates the background fluorescence signal from the diffusing imager as well as improves the imaging speed to a significant extent (Figure 2A). Recently, Uno et al. implemented this approach using spiro cyclization‐based fluorogenic rhodamine dyes [38]. They have developed a series of such dyes which exist in two states: closed form and open form. In solution, these dyes remain in closed form which is nonfluorescent and then, upon binding through a short peptide that can transiently associate with its cognate protein target, it gets converted into the fluorescent open form. As a result, the binding–unbinding events lead to fluorescence blinking at the target sites. The blinking kinetics are determined by the rate of association (*k*_on_) and dissociation (*k*_off_) between the aforementioned peptide and the target, resulting in no need of photo‐switching fluorophore and high localization precision (5–10 nm) of individual single‐molecule images.

Moliner et al. developed a library of peptide‐based fluorogenic probes that eliminates the necessity to incorporate chemical spacers between the probe and the peptide, so that any potential impact of the probe on the biophysical properties of the imager can be minimized. In solution, benzodiazole amino acids keep the binding capabilities of bioactive peptides intact. This enables background‐free, wash‐free peptide‐guided imaging. These artificial amino acids are highly sensitive to the environment, have high photo‐stability, and are fully compatible with solid‐phase peptide synthesis. They have incorporated these amino acids into the transiently binding peptide pairs that make these imager peptides fluorescent only upon engaging with the target. Hence, these are called self‐reporting Peptide‐PAINT imagers. This approach has been employed to map endogenous postsynaptic density protein‐95 (PSD95) nanoclusters in mouse brain synaptosomes from mouse brain tissues and achieved high signal‐to‐noise ratio. This work establishes fluorogenic amino acids as minimal, genetically agnostic reporters that substantially improve specificity as well as the imaging speed of Peptide‐PAINT [37].

Weiss et al. developed fluorogenic peptide probes by conjugating the environmentally sensitive dye, Nile Red to an antimicrobial peptide, Ubiquicidin, enabling wash‐free and background‐suppressed imaging. By systematically varying the conjugation site and chemistry, they have demonstrated that internal incorporation of Nile Red derivative as a minimalistic unnatural amino acid (UNR‐1) yields superior fluorogenic response and target specificity compared to terminal or side‐chain labeling (Figure 2A). The probes remain weakly fluorescent in solution but switch on upon binding to bacterial membranes, allowing add‐and‐read detection (Figure 2B). Single‐molecule blinking arising from transient peptide binding enables super‐resolved reconstruction of bacterial envelopes, while line‐profile and full width at half maxima (FWHM) analysis confirm membrane‐localized signal distinct from intracellular DNA staining. Together, these results establish rational fluorophore placement within peptides as a key design principle for FPP, enabling high‐contrast, minimally perturbative super‐resolution imaging (Figure 2C). However, we must note that such an approach is applicable to image very specific targets [39].

Overall, there is a significant progress of FPP that aids to minimize the background signal from the unbound diffusing imagers, improves signal‐to‐noise ratio at similar concentrations of imager in comparison to conventional Peptide‐PAINT, and also, allows to use higher concentrations of imager, resulting increase in the rate of binding of imagers which ultimately improves the rate of data acquisition. However, many currently available fluorogenic systems remain target‐specific and require careful molecular engineering, limiting their applicability. Thereby, it is pertinent to develop universally applicable fluorogenic peptide probes with predictable photo‐physical and kinetic properties.

### 3.3 Endogenous Peptide‐PAINT (EPP)

**FIGURE 3:**
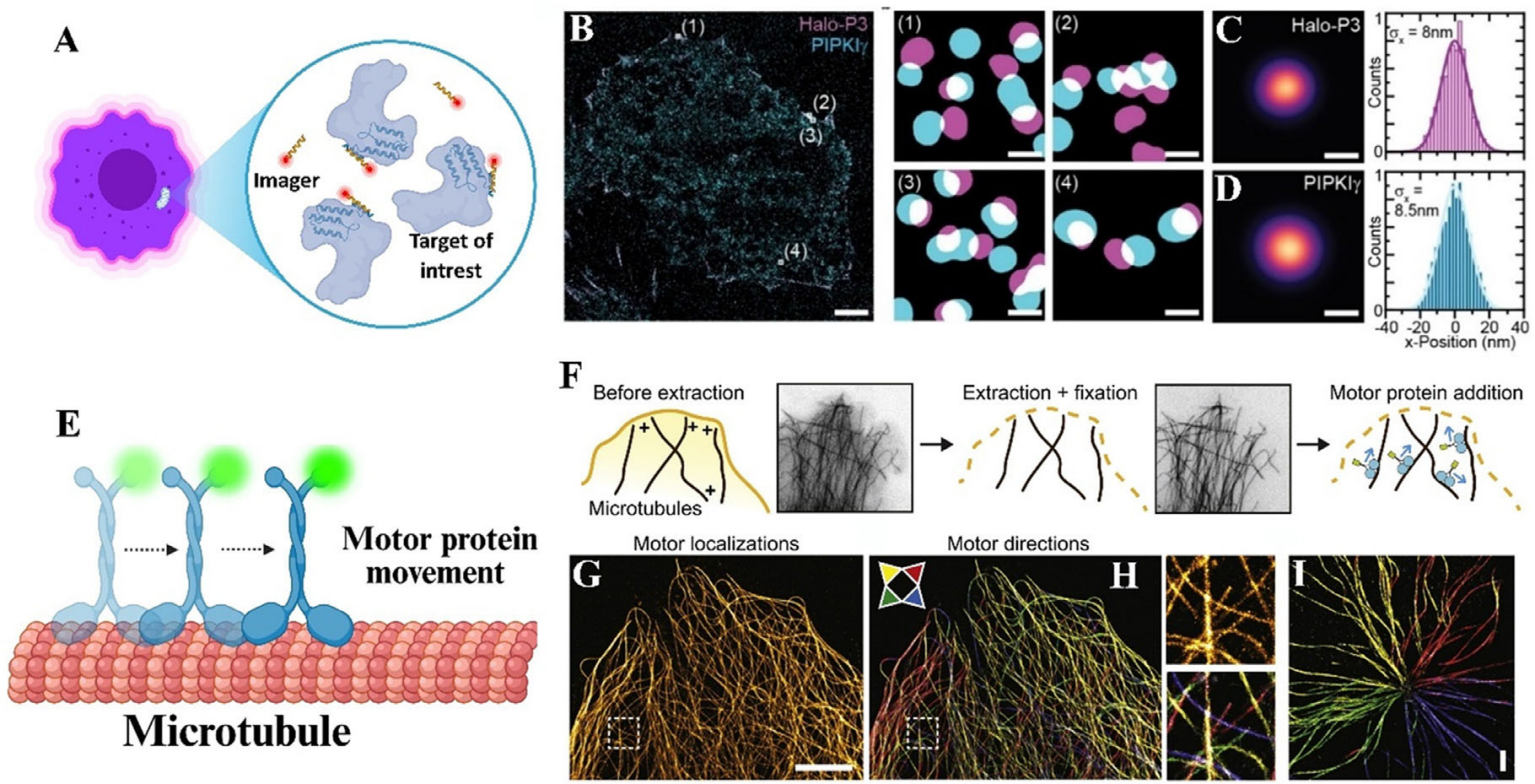
(A) Schematic representation of endogenous PAINT‐based visualization of target proteins; (B) dual‐color super‐resolution image, showing nanoscale localization and colocalization of Halo‐P3 and PIPKIγ‐labeled proteins; scale bar: 2 µm; localization precision distributions of individual single molecule events during DNA‐PAINT (C) and Peptide‐PAINT (D) imaging. Scale bar: 40 nm; (E) illustration of fluorophore‐tagged motor protein movement along the microtubules; (F) experimental workflow for extraction, fixation, and motor protein addition to map microtubule polarity and organization; (G) super‐resolution reconstruction of motor localizations on microtubule networks; scale bar: 5 µm; (H) directionality mapping of motor trajectories revealing polarized microtubule organization and filament connectivity; (I) color‐coded visualization of motor transport pathways with direction; scale bar: 1 µm (Adapted from Tas et al., Neuron, 2017 & Fischer et al., ChemBioChem, 2021).

Despite Peptide‐PAINT being a powerful tool to obtain highly resolved images of target proteins in cells, it has certain disadvantages in terms of accuracy as well as biological relevance [39]. Conventional Peptide‐PAINT suffers from considerable linkage error due to use of primary and secondary antibodies as intermediate labeling agents of a docker peptide. On the other hand, engineered docking systems mandates recombinant expression of the docker with the target proteins which is usually much higher than the physiological expression level [25], resulting in dysfunction, mis‐localization, interference in protein–protein interaction or dynamic assemblies. Thereby, imaging an endogenous target protein using PAINT, termed as EPP is very crucial. EPP does not use any engineered docking systems, rather replaces them with direct and reversibly recognizing peptide/protein to a particular domain of a protein (Figure 3A). For an example, Fischer et al. recently showed that a Cy3b‐labeled imager peptide (Table 1), derived from phosphatidylinositol (4) phosphate 5 kinase type Iγ, transiently binds to Talin, a key mechanosensitive protein involved in cell adhesion at the extracellular interface, and allows to visualize at the molecular scale [40]. This approach combines the principles of Peptide‐PAINT and image reconstruction by integrating exchangeable single‐molecule localization (IRIS) methodology, which employs exchangeable protein fragment–based probes that reversibly associate and dissociate from their targets on millisecond timescales, generating stochastic fluorescence signals suitable for PAINT‐based SMLM. This enabled quantitative analysis of Talin in its native cellular environment without permanent labeling. They got nearly the same imaging pattern and localization as DNA‐PAINT (Figure 3B,C,D). This direct binding strategy substantially reduces linkage error and improves localization accuracy.

Tas et al. introduced an innovative strategy to implement exchange‐based super‐resolution imaging for probing endogenous microtubule organization in neuronal processes [41]. In this approach, fluorescently labeled variants of motor proteins, including Kinesin‐1 and Kinesin‐3, were externally applied to chemically fixed and permeabilized neurons, where they retained the ability to translocate along microtubules (Figure 3E,F). By tracking the nanometric movement of these plus‐end‐directed motors, a large number of single‐molecule localization events were accumulated, enabling reconstruction of microtubules with high spatial resolution (FWHM ≈ 52 ± 2 nm) along with direct determination of filament polarity (Figure 3G). This method, termed *“*Motor‐PAINT,” revealed that dendritic microtubules are organized into biochemically distinct bundles with opposite orientations (Figure 3H,I). Notably, Kinesin‐1 preferentially associates with stable, acetylated, minus‐end–out microtubules, whereas Kinesin‐3 selectively binds dynamic, tyrosinated, plus‐end–out microtubules, providing a mechanistic basis for their differential transport behaviors. More broadly, this work establishes that reversible, endogenous‐like biomolecular interactions can be harnessed to drive PAINT imaging, offering a conceptual framework for developing minimally perturbative, biologically specific EPP strategies [41].

The concept of exchangeable probe‐based imaging (EPP) has been successfully extended to encompass additional classes of biomolecules, notably glycans, under physiologically relevant conditions. This development, broadly termed Glyco‐PAINT, facilitates the visualization of the nanoscale organization of cell‐surface glycans—including those associated with receptors, the glycocalyx, and proteoglycans—within their native cellular context. In this framework, Tholen et al. introduced Lectin‐PAINT, also referred to as Glyco‐PAINT, as a super‐resolution strategy that exploits the transient, low‐affinity interactions between lectins and glycans. By utilizing a series of fluorescently labeled lectins with distinct carbohydrate specificities, they have demonstrated multiplexed PAINT imaging of the cellular glycocalyx without the need for covalent modification of glycan structures. The reversible lectin–glycan interactions give rise to stochastic fluorescence “blinking,” enabling single‐molecule localization and tracking in live‐cell environments. Notably, this approach permits the extraction of spatial distribution, binding kinetics, and molecular mobility, thereby enabling the generation of a quantitative, single‐cell “glycotype.” Collectively, these findings establish glycan–lectin interactions as an effective endogenous contrast mechanism for PAINT‐based imaging, extending the principles of Peptide‐PAINT to complex carbohydrate landscapes. Such direct visualization of glycans at the single‐molecule level holds considerable promise for early and precise disease diagnostics, as it enables detection of subtle nanoscale alterations in glycosylation patterns across diverse pathological conditions, including cancer, inflammation, infection, neurodegeneration, congenital glycosylation disorders, and fibrosis [42, 43].

Farrell et al. developed pPAINT, a super‐resolution imaging approach that repurposes native protein–protein interaction domains as transient PAINT probes. By employing fluorescently labeled SH2 domains (tSH2 domain of Zap70, syk kinase, SHP2), they have directly visualized phosphor‐tyrosine signaling sites at the T‐cell plasma membrane without relying on antibodies or genetic modification. The reversible SH2–phosphor‐tyrosine interactions produced stochastic binding and unbinding events, generating the blinking required for SMLM. Beyond nanoscale spatial mapping, pPAINT enabled in situ quantification of binding kinetics, including dissociation half‐lives within individual signaling clusters. This study established endogenous interaction domains as functional PAINT probes, extending Peptide‐PAINT toward a minimally perturbative and multiplex platform with strong potential for diagnostic applications, such as detecting disease‐specific signaling molecules and pathological states [44].

### 3.4 Multiplexed Peptide‐PAINT

**FIGURE 4:**
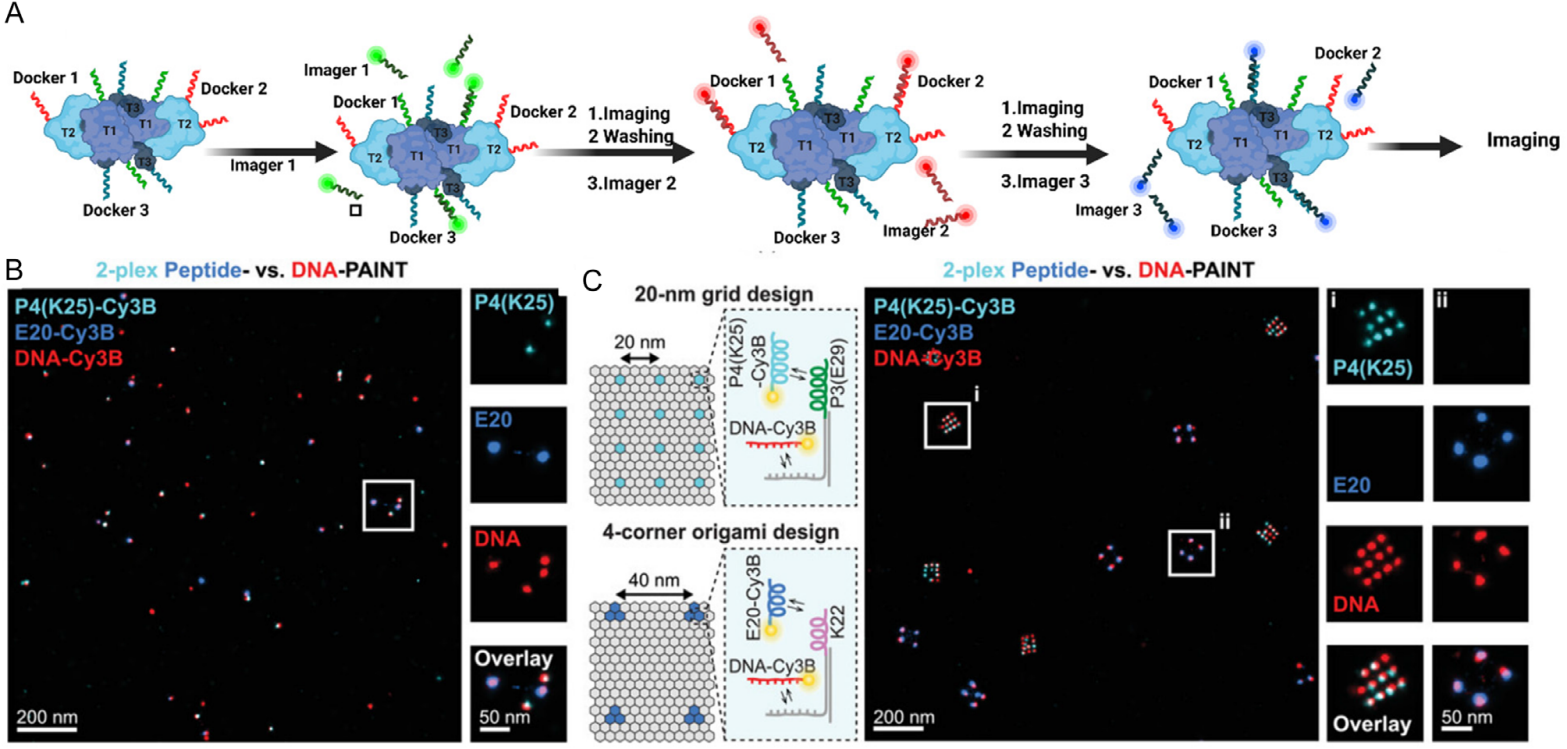
(A) Schematic of multiplex Peptide‐PAINT imaging using sequential addition of different imager peptides one at a time followed by washing; (B) 2‐plex Peptide‐PAINT with P4(K25)‐Cy3B (cyan) and E20‐Cy3B (blue), showing orthogonal binding and DNA‐PAINT colocalization (red); (C) two DNA origami architectures for the orthogonality assay (left). Two‐plex Peptide‐PAINT with P4(K25)‐Cy3B and E20‐Cy3B (center). Insets show the 20‐nm grid (i) and 4‐corner structure (ii) (right). P4(K25) labels only (i), E20 only (ii), with DNA‐PAINT colocalization (red) (Adapted from Eklund et al., Small, 2023).

Peptide‐PAINT has established itself as a minimally perturbative yet highly effective approach for super‐resolution imaging of proteins, with a notable strength in its capacity for multiplexed detection. Conceptually analogous to DNA‐PAINT, multiplexed implementations of Peptide‐PAINT enable the simultaneous interrogation of multiple molecular targets within a single specimen, thereby providing detailed insights into nanoscale spatial organization, co‐localization, and interaction networks. This capability is achieved through the use of orthogonal docker–imager interactions, in which each imager peptide selectively recognizes its corresponding docker with minimal cross‐reactivity. Imaging is typically performed in a sequential manner, allowing repeated rounds of target visualization without compromising sample integrity (Figure 4A). By designing multiple orthogonal docker–imager pairs with distinct sequences and binding affinities, different targets can be independently resolved within the same sample while maintaining distinguishable stochastic binding kinetics. In this context, Eklund et al. demonstrated 2‐plex Peptide‐PAINT imaging using two orthogonal coiled–coil systems, namely the E20–K22 and P3–P4 pairs (Table 1). The E20–K22 pair was derived from modified E/K coiled–coil motifs, whereas the P3–P4 pair exhibited one of the most specific and robust interactions among previously de novo designed P1–P12 variants. Orthogonality between these systems was validated using DNA origami‐based in vitro assays (Figure 4B). The transient yet highly specific binding interactions enable efficient washing and reintroduction of imagers, thereby minimizing cross‐reactivity. The reported binding lifetimes (~234 ms for E20–K22 and ~259 ms for E20–P4(K25)) are well‐suited for SMLM (Table 1). Importantly, this strategy achieved cross‐talk levels below 2%–5% while maintaining a localization precision of 3–6 nm, highlighting the robustness of Peptide‐PAINT for high‐content nanoscale imaging. However, these orthogonal peptide pairs have not yet been directly applied to resolve nanoscale architectures of biomolecules. Instead, a hybrid approach combining Peptide‐PAINT and DNA‐PAINT was employed to perform 2D and 3D 2‐plex imaging of human epidermal growth factor receptor 2 (ErbB2/HER2) in fixed CHO cells, enabling the resolution of extracellular and intracellular domains separated by 11–25 nm [26].

Beyond sequential multiplexing, alternative strategies such as spectral and kinetic multiplexing have also been explored. Spectral multiplexing relies on the use of distinct fluorophores conjugated to different imagers, enabling simultaneous detection of multiple targets [45]. However, its scalability is constrained by spectral overlap and chromatic aberrations, which can compromise resolution. Approaches leveraging Förster resonance energy transfer (FRET) efficiencies have also been proposed to enhance spectral multiplexing in PAINT imaging [46]. Consequently, spectral strategies are often combined with sequential exchange methods to expand multiplexing capacity. Kinetic multiplexing represents a more recent and conceptually distinct approach, in which targets are differentiated based on their binding kinetics rather than spectral signatures. In this framework, the dwell time of binding events is tuned by modulating interaction strength (e.g., number of interacting residues), while binding frequency is controlled by the number of available binding motifs. As these parameters are orthogonal, they can be combinatorically encoded to create a scalable and information‐rich multiplexing scheme. Notably, such kinetic encoding strategies have thus far been primarily demonstrated in DNA‐PAINT systems [47] and remain to be fully explored in the context of Peptide‐PAINT.

### 3.5 Live‐Cell Peptide‐PAINT

A central challenge in cell biology is the ability to resolve the nanoscale organization and dynamics of biomolecules within living cells. While DNA‐PAINT has emerged as a powerful super‐resolution modality, its application to live‐cell imaging is limited by the difficulty of conjugating DNA‐docking strands to intracellular targets and delivering fluorescent imager strands across the cell membrane. In this context, Peptide‐PAINT offers a compelling alternative, particularly when combined with genetically encoded labeling strategies. For example, Oi et al. demonstrated live‐cell Peptide‐PAINT imaging of the septin protein Cdc12p in Saccharomyces cerevisiae [36]. In their study, two transiently interacting peptide pairs—TRAP4–MEEVF and SYNZIP17–SYNZIP18—were identified, exhibiting dissociation constants of ~300 nM and ~1 nM, respectively (Table 1). TRAP4 or SYNZIP17 was fused to the fluorescent protein mNeonGreen (mNG), while the corresponding docker peptides (MEEVF or SYNZIP18) were genetically appended to the target protein. Both the docker‐tagged targets and the fluorescent imagers were encoded within the cell, with imager expression placed under an inducible promoter to enable precise control over expression levels for optimal single‐molecule detection. Transient and reversible nature of the peptide interactions enabled continuous replenishment of fluorescence signals, thereby reducing photo‐bleaching rate and allowing extended imaging durations. Notably, this approach has enabled live‐cell Peptide‐PAINT imaging with a spatial resolution of ~20 nm. Using this strategy, the nanoscale organization of Cdc12p was quantitatively analyzed, revealing that the septum width at the bud neck remains relatively constant until the daughter cell diameter reaches approximately 0.85 of the mother cell diameter, beyond which the septum resolves into two distinct rings. Overall, this genetically encoded implementation significantly simplifies sample preparation by eliminating the need for fixation, permeabilization, blocking, and exogenous labeling. However, the reliance on fluorescent proteins—typically less bright than organic fluorophores used in SMLM—results in reduced localization precision. Furthermore, the limited intracellular pool of imager molecules leads to fast decrease in localization events over time, potentially causing undersampling and limiting the resolution of nanoscale structural features [48].

Expanding the applicability of Peptide‐PAINT to living neuronal systems, Maity et al. demonstrated that imaging surface proteins using a transfected docker and an external imager appended to a bright organic fluorophore. They have employed this technique to investigate the nanoscale organization of synaptic proteins, including GluA2 and neuroligin, in living neurons. GluA2, a subunit of AMPA receptors, and neuroligin, a postsynaptic adhesion protein interacting with neurexin, were imaged using the E22–K19 coiled–coil docker–imager pair (Table 1). In this strategy, the E22 docker was genetically fused to the target proteins, thereby circumventing complex chemical‐labeling procedures and minimizing linkage errors. This approach enabled localization precision approaching ~10 nm. Super‐resolution imaging further revealed that AMPA receptors are organized into nanoscale domains in proximity to the synaptic cleft despite their lateral mobility within the postsynaptic membrane. In contrast, neuroligin exhibited a comparatively broader spatial distribution, with a substantial fraction localized ~500 nm away from postsynaptic regions [25].

**FIGURE 5:**
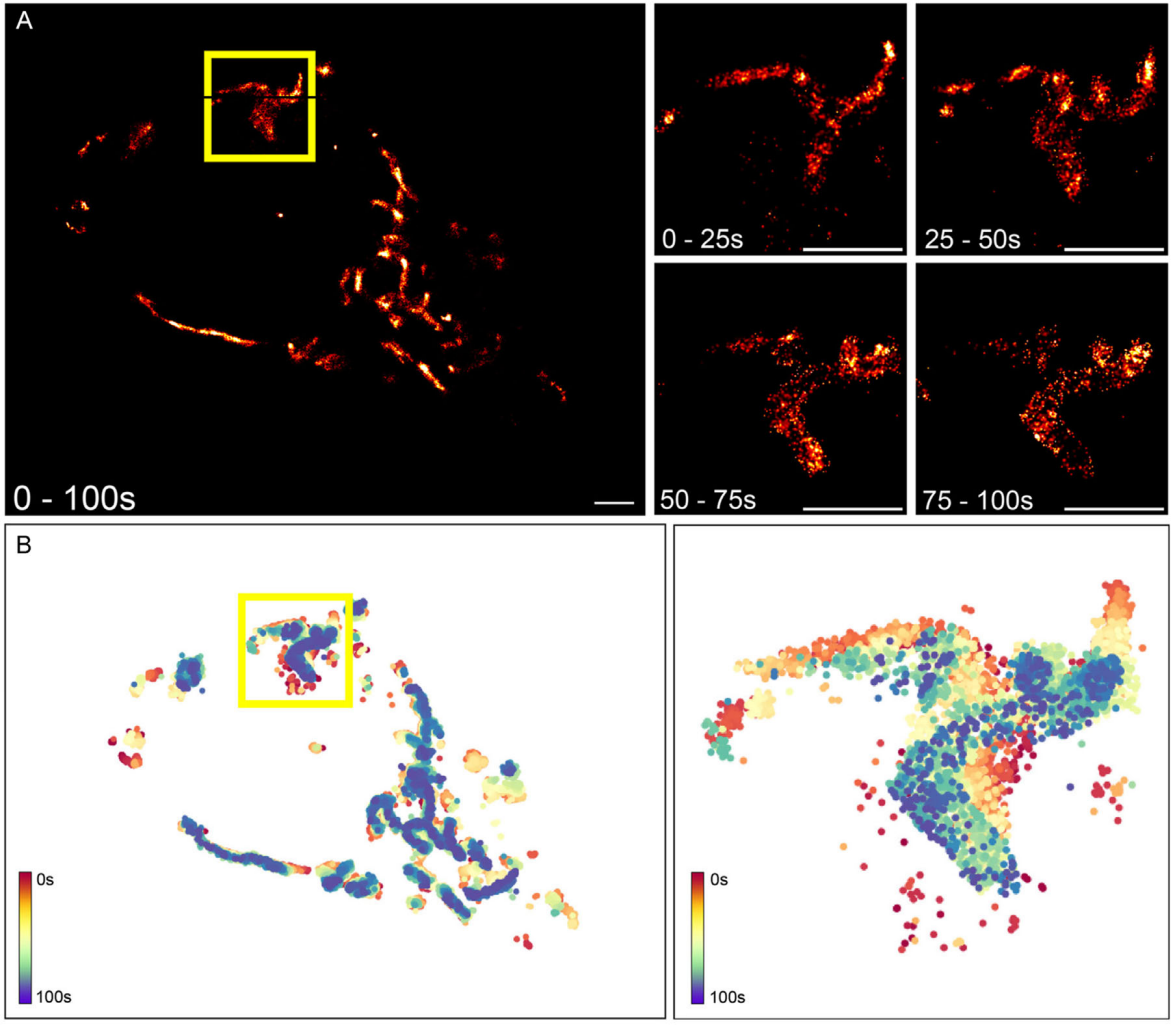
(A) Super resolution images of TOM20 imaged for 100 s. The subsequent images (right) are the ROI‐reconstructed images in particular time with the 100 s. (B) Localizations obtained from the entire field of view (FOV) and the selected region of interest (ROI) during the 100‐s imaging interval, color‐coded according to the temporal sequence of acquisition from 0 to 100 s (scale bar: 2 µm). (Adapted from Bhasker et al., Protein Sci., 2025).

More recently, Bhaskar et al. developed mLIVE‐PAINT, a peptide‐based SMLM platform specifically designed for live mammalian cells. This strategy exploits the reversible interaction between engineered coiled–coil peptides, 101A and 101B, to achieve transient fluorescent labeling without directly attaching bulky fluorophores to the protein of interest (Table 1). Such a minimally invasive labeling strategy reduces perturbation to endogenous protein localization and function. Using mLIVE‐PAINT, the authors achieved nanoscale imaging of the mitochondrial protein TOM20 and nuclear protein Histone H2B with the spatial resolution of ~60 nm (Figure 5A). Furthermore, they have been able to capture spatiotemporal dynamics of mitochondrial networks in living cells (Figure 5B). Collectively, these studies highlight the versatility of Peptide‐PAINT‐based methodologies as minimally perturbative tools for investigating protein organization and dynamics in live‐cell environments [34].

Live‐cell Peptide‐PAINT arguably represents one of the most distinctive advantages of peptide‐based PAINT approaches over DNA‐PAINT because genetically encoded peptide dockers simplify intracellular targeting while preserving transient probe exchange. However, current implementations remain limited by fluorescent protein brightness, imager concentration, and potential perturbations associated with recombinant protein expression. Future advances will require minimally invasive endogenous labeling strategies combined with brighter fluorogenic probes and optimized peptide engineering to fully realize long‐term live‐cell super‐resolution imaging.

## 4 Comparison With Other SMLM Approaches

While STORM and PALM, widely used single‐molecule imaging‐based super‐resolution approaches, utilize photo‐physical switching behavior of fluorophore photo‐physics to generate fluorescence blinking, PAINT achieves this using transient binding–unbinding kinetics between the docker and the imager as discussed above. This fundamental difference provides several technical advantages. For example, due to rapid exchange of imagers at the target sites and exposure of excitation light at a specific region of the specimen, which was commonly achieved by HILO excitation [32], effective rate of photo‐bleaching as well as fluorescence background from out‐of‐focus regions get substantially reduced. As a result, PAINT maintains a steady supply of imagers throughout the experiment, enabling the acquisition of a larger number of single‐molecule localizations for a longer period of time unlike STORM and PALM (Table 2). The increased sampling density improves the ability to resolve fine nanoscale structures.

**TABLE 2:** Comparison between different single‐molecule imaging‐based super‐resolution techniques.

Technique	Localization precision	Multiplexity	Biocompatibility	Key references
PALM	10–30 nm	Moderate (limited by photoactivatable fluorophores)	High in live‐cell imaging	Jesen et al., 2022; Kikuchi et al., 2022
STORM	10–20 nm	High (multiple dye combinations possible)	Moderate (often requires special buffers and dyes)	Tam et al., 2015; Gomez‐Garcia et al., 2018; Klevanski et al., 2020
DNA‐PAINT	1–10 nm	Very high (sequence‐programmable multiplexing)	Moderate to low for live‐cell imaging (mostly fixed‐cell compatible)	Schnitzbauer et al., 2017; Jungmann et al., 2014; Stein et al., 2019; Deußner‐Helfmann et al., 2018., Ostershelt et al., 2022.
Peptide‐PAINT	5–20 nm	High	Higher than DNA‐PAINT for live‐cell applications	Eklund et al., 2020; Tas et al., 2021; Maity et al., 2023; Fischer et al., 2021

PAINT offers remarkable capabilities for multiplexed imaging, stemming from its unique reliance on binding–unbinding kinetics to generate fluorescence blinking. In contrast to conventional SMLM approaches, which are fundamentally constrained by the limited number of spectrally distinct blinking fluorophores that can be simultaneously resolved, PAINT‐based methods decouple multiplexing from spectral bandwidth. Instead, multiplexing is primarily determined by the availability of orthogonal docker–imager pairs. This feature enables the use of distinct binding sequences to label different molecular targets, allowing either sequential imaging through exchange of imager strands or, in certain implementations, simultaneous detection strategies. As a result, PAINT significantly expands the multiplexing capacity beyond traditional limits, facilitating the visualization of multiple biomolecular species within the same sample. Such scalability is fundamentally applicable for both DNA‐PAINT and Peptide‐PAINT [22, 49]. These are particularly advantageous for studying complex biological systems, where the ability to map numerous interacting components at nanometer resolution provides deeper insights into molecular organization and function. However, multiplexed PAINT imaging has thus far been predominantly demonstrated using DNA‐PAINT, whereas its implementation in Peptide‐PAINT remains comparatively limited. This disparity largely stems from the limited availability of well‐characterized, mutually orthogonal docker–imager pairs, which currently represents a major bottleneck for multiplexed Peptide‐PAINT imaging and is discussed in detail in a later section.

Despite several advantages, DNA‐PAINT has certain limitations. A key challenge arises from the requirement to attach DNA‐docking strands to target molecules using labeling agents, which can introduce significant linkage error and potentially perturb the native spatial organization of proteins. Such modifications may be particularly problematic when probing nanoscale architectures, where even small positional offsets can impact quantitative interpretation. To address these limitations, alternative strategies employing small peptide pairs have been developed, giving rise to Peptide‐PAINT. In this approach, transient interactions between peptide tags and their binding partners replace DNA hybridization, thereby reducing the overall steric footprint of the labeling system. By leveraging native or engineered peptide–protein interactions, Peptide‐PAINT maintains the fundamental advantages of the PAINT methodology—including continuous probe replenishment, resistance to photo‐bleaching, and high localization precision—while offering improved biological compatibility and more accurate representation of molecular organization.

DNA‐PAINT relies on transient DNA hybridization and therefore, requires imaging buffers with relatively high salt concentrations to stabilize duplex formation and control binding kinetics [50]. In contrast, Peptide‐PAINT is compatible with physiological buffers such as PBS or standard cell‐culture media, making it better suited for imaging under native and live‐cell conditions [23]. The imaging probes also differ significantly. DNA imagers are highly negatively charged oligonucleotides that could exhibit nonspecific adsorption to positively charged cellular components, membranes, extracellular matrix molecules, and inadequately passivated surfaces, leading to increased background as well as nonspecific localizations [51]. In contrast, peptide imagers are smaller and diffuse more readily in crowded cellular environments, although their nonspecific interactions depend on sequence composition. By optimizing amino acid sequence, charge distribution, and hydrophobicity, peptide probes generally exhibit lower nonspecific binding [52] and improved target accessibility compared to DNA imagers in complex biological samples.

Additionally, in the context of quantitative molecular imaging, qPAINT offers several conceptual advantages over photo‐switching‐based SMLM approaches, including STORM and PALM. Quantification using STORM or PALM fundamentally relies on the complex photo‐physical behavior of blinking or photoactivatable fluorophores and therefore, often requires careful characterization of multiple kinetic parameters to distinguish repeated emissions from the same fluorophore versus signals originating from distinct molecules. Accurate determination of these parameters can be experimentally challenging and may introduce substantial uncertainty into molecular counting [53]. Moreover, spatially nonuniform illumination, variations in excitation or activation laser intensity, and progressive photo‐bleaching can alter fluorophore‐blinking statistics and detection efficiency, further complicating absolute quantification. In contrast, qPAINT derives molecular abundance from the stochastic association kinetics between docking sites and freely diffusing imagers as discussed above, and is therefore, comparatively less dependent on fluorophore photo‐physics as well as photo‐bleaching behavior [12]. Nevertheless, qPAINT is not inherently free from quantitative bias, as variations in docking‐site accessibility, and local association kinetics can directly influence the measured dark‐time distributions [12]. Thus, altogether, qPAINT provides a potentially more robust kinetic framework for molecular counting than direct localization‐based STORM or PALM approaches, but rigorous calibration and validation of binding kinetics remain essential for accurate quantitative imaging.

## 5 Current Challenges and Limitations of Peptide‐PAINT

Despite transformative impact on super‐resolution imaging, Peptide‐PAINT faces several technical and conceptual challenges that limit its broader applications in biological systems. For example, conventional Peptide‐PAINT imaging uses intermediate labeling agents to attach the docker peptide to the target of interest as discussed above. While such approach enables imaging under an endogenous environment, it introduces significant linkage error, often exceeding single‐molecule localization precision, which limits the resolution. Hence, this becomes particularly problematic when resolving densely packed nanoscale structures or probing protein–protein interactions in crowded environments. Although genetic fusion of docker to the target proteins reduces the linkage error, this approach is not applicable to endogenous proteins. More broadly, these considerations highlight that the biological compatibility of Peptide‐PAINT is not solely determined by the use of peptide probes but also by the associated labeling strategy. While genetically encoded docking systems reduce linkage error and simplify probe delivery, they may introduce biological perturbations arising from recombinant protein expression or peptide fusion. Therefore, the development of endogenous labeling strategies, minimally invasive peptide tags, and genome‐editing‐based approaches are essential to enable imaging of endogenous proteins while improving the physiological relevance of Peptide‐PAINT.

One of the major advantages of PAINT in comparison to other single molecule imaging‐based techniques is the higher multiplexing ability. However, the multiplexing capability of Peptide‐PAINT is constrained by the limited availability of orthogonal docker–imager pairs. In contrast to DNA, designing peptide binders with high specificity, appropriate transient‐binding kinetics, and minimal cross‐reactivity remains challenging. Additionally, nonspecific interactions in complex biological environments further reduce the number of targets that can be imaged simultaneously. In this context, emerging approaches based on machine learning and computational design hold promise for generating novel peptide pairs with tunable kinetics and high specificity, thereby enabling simultaneous visualization of multiple targets at nanoscale resolution.

The fundamental mechanism of Peptide‐PAINT—stochastic binding and unbinding events that create fluorescence blinking—also introduces a tradeoff between imaging speed and background noise. Increasing the concentration of freely diffusing imagers enhances the imaging speed, and thus accelerates data acquisition. However, higher imager concentrations also elevate background fluorescence, ultimately limiting achievable imaging speed and signal‐to‐noise ratio. The development of fluorogenic probes offers a potential solution but remains an area of active research.

Extending Peptide‐PAINT to live‐cell imaging presents additional challenges. Most current implementations are performed in chemically fixed samples, where molecular motion is restricted. In living cells, factors such as diffusion of target proteins, degradation of imager, and cellular toxicity significantly compromise both spatial and temporal resolution. Moreover, efficient intracellular delivery of imager peptides remains difficult. Imagers could be trapped in endosomes or degraded before reaching the targets of interest.

Steric accessibility poses a significant limitation in crowded biological environments. In structures such as biomolecular condensates or densely packed cytoskeletal networks, the docker appended to the target of interest may become partially or completely occluded. As a result, imager binding is restricted to exposed regions, leading to systematic undersampling of buried populations. This steric shielding can produce apparent heterogeneity that does not reflect the true molecular organization.

Although the fundamental concept of qPAINT is very intriguing, extending this approach with Peptide‐PAINT is very challenging in comparison to DNA‐PAINT. DNA hybridization offers predictable association and dissociation rates that can be adjusted by changing the sequence length, GC content, ionic strength, and temperature [10]. In contrast, peptide–protein recognition relies on a set of factors such as electrostatic interactions, hydrogen bonding, hydrophobic contacts, flexibility, and steric accessibility [52]. As a result, peptide‐docking interactions often show target‐dependent variations in kinetics, leading to significant differences in both *k*_on_ and *k*_off_, even among the same proteins in different cellular environments.

## 6 Future Perspectives

Peptide‐PAINT has been established to be a very powerful tool in super‐resolution microscopy, and its future development is poised to significantly increase its applicability in quantitative and functional biology. Continued advances in probe design, labeling strategies, and imaging integration tools are expected to transform Peptide‐PAINT from a primary spatial imaging tool into a versatile platform for probing complex biological systems.

A key future direction lies in the development of sequential and highly multiplexed imaging strategies. By leveraging a set of orthogonal docker–imager pairs, Peptide‐PAINT can enable the visualization of multiple proteins within the same sample at nanoscale resolution. Such multiplexing will be critical for unraveling the spatial organization of complex protein networks underlying diverse cellular functions, including signaling assemblies, transcription regulation, and biomolecular condensates.

Another important application avenue is the development of EPP, which would eliminate the need for overexpression of target proteins or bulky labeling intermediates. Imaging proteins at their native expression level will not only improve effective spatial resolution by minimizing linkage errors but also preserve physiological stoichiometry and interaction network. This advancement will position Peptide‐PAINT as a robust tool for quantitative nanoscale cell biology, enabling accurate measurements of molecular distributions and interactions in their native context.

In the future, combining Peptide‐PAINT with complementary imaging modalities will further broaden its scope. Integration with techniques such as fluorescence lifetime imaging [54], single particle tracking, and correlative light‐electron microscopy [55] will allow simultaneous mapping of structure, dynamics, and molecular interactions. Such multimodal approach has the potential to transform Peptide‐PAINT into a comprehensive functional‐imaging platform, transforming Peptide‐PAINT from a purely spatial technique into a comprehensive functional‐imaging platform bridging the gap between structural mapping and mechanistic understanding.

Beyond these developments, recent advances in computational peptide design and artificial intelligence are expected to play a transformative role in the next generation of Peptide‐PAINT technologies. Furthermore, integration of fluorogenic peptide imagers with advanced localization software (e.g., Picasso [10], ThunderSTORM [56], and SMAP [57]) and next‐generation AI‐driven SMLM approaches—including deep learning‐assisted localization [58], point‐cloud reconstruction [59], spectroscopic analysis [60], and quantitative image interpretation—is expected to substantially enhance spatial resolution, multidimensional information extraction, and quantitative biological analysis, thereby expanding the capabilities of Peptide‐PAINT for high‐content super‐resolution imaging, will accelerate image acquisition and reconstruction, and quantitative analysis of densely labeled biological samples [61]. Collectively, these technological innovations will establish Peptide‐PAINT as a powerful platform for quantitative biology, enabling comprehensive investigation of molecular interactions, signaling networks, and spatially organized cellular processes across multiple spatial and temporal scales. Further BGnet‐based background estimation will enable faster, more accurate single‐molecule localization, improving image quality, localization precision, and temporal resolution in future SMLM experiments [62].

Finally, advancements in intracellular delivery strategies, peptide stability, and overall biocompatibility will extend the application of Peptide‐PAINT to more complex biological systems such as intact tissues, organoids, and in vivo systems. Such progress will strengthen the bridge between high‐resolution microscopy and physiological relevance, enabling nanoscale investigations directly within intact biological environments.

## 7 Conclusion

Peptide‐PAINT represents a significant development in the field of super‐resolution microscopy by combining the conceptual simplicity of PAINT with the biological relevance and versatility of peptide‐based interactions. This method attains high localization precision, low linkage error, photo‐bleaching resistance, and compatibility with quantitative and multiplexed imaging by using transient, programmable peptide target binding. The inventions of fluorogenic, endogenous, multiplexed, and live‐cell Peptide‐PAINT, among others, are proof of its versatility in different biological contexts. Despite several challenges related to ON–OFF kinetics, delivery of imager into living cells, multiple orthogonal docker–imager pairs, it represents a better comparative imaging scenario in comparison with other available super‐resolution methods.

## Author Contributions

B.K.M**.** conceptualized the idea. D.P.B., R.S., A.G., and B.K.M**.** co‐wrote the manuscript.

## Acknowledgments

We acknowledge the support of Saha Institute of Nuclear Physics, Kolkata; Homi Bhabha National Institute (HBNI), Mumbai; and Department of Atomic Energy, India. B.K.M also acknowledges the financial support from the Early Career Research Grant (ECRG) of the Science and Engineering Research Board (SERB), Government of India (Grant No.: ANRF/ECRG/2024/001401/CS), and the One Nation One Subscription (ONOS) Initiative of Govt. of India (https://ror.org/036h6g940).

## Funding

This work was supported by the ANRF (ANRF/ECRG/2024/001401/CS) and the Department of Atomic Energy, Government of India (RSI 4008). The Open Access Fees for this article is fully funded by the One Nation One Subscription (ONOS) Initiative of Govt. of India (https://ror.org/036h6g940).

## Conflicts of Interest

The authors declare no conflicts of interest.

## Data Availability

Data sharing is not applicable to this article as no datasets were generated or analyzed during the current study.
